# Cross-cultural Psychometric Evaluation of the Dutch McGill-QoL Questionnaire for Breast Cancer Patients

**Published:** 2016-12

**Authors:** T De Vrieze, D Coeck, H Verbelen, N Devoogdt, W Tjalma, N Gebruers

**Affiliations:** Department of Rehabilitation Sciences and Physiotherapy-MOVANT, Faculty of Medicine and Health Sciences, University of Antwerp, Universiteitsplein 1, 2610 Antwerp, Belgium; Department of Rehabilitation Sciences, Tervuursevest 101, box 1500, B-3001 Heverlee, Belgium; Multidisciplinary Breast Clinic, Antwerp University Hospital (UZA), Wilrijkstraat 10, 2650 Edegem, Belgium; Department of Medicine, Faculty of Medicine and Health Sciences, University of Antwerp, Universiteitsplein 1, 2610 Antwerp, Belgium

**Keywords:** Breast cancer, mcgill-qol questionnaire, reliability, validity

## Abstract

**Aim of the study:**

Assessing the cross-cultural psychometric properties of the Dutch-MQoL for breast cancer patients.

**Methods:**

26 patients were recruited at the Antwerp University Hospital. Eligible patients filled in the MQoL on different moments in time in order to evaluate clinimetric properties. To determine the validity; MQoL was correlated to the EORTC QLQ-C30. Internal consistency was analysed using Cronbach’s and test-retest reliability was determined by ICC. For statistical responsiveness, S.E.M and MDC were calculated.

**Results:**

A strong correlation was found between the ‘QoL score’ of the MQoL and the domain ‘existential well- being’ of the EORTC QLQ-C30 (r = 0.72). An excellent test-retest reliability (ICC (1,1)) was demonstrated with intraclass coefficients ranging from 0.82 to 0.92. A MDC in total score of only 1.22 (12%) was seen, needed to detect a factual change within a patients’ QoL. Psychometric properties of the Dutch MQoL were found comparable to the properties of the original questionnaire.

**Conclusion:**

The Dutch version of the MQoL is a valid and reliable questionnaire for breast cancer patients and shows statistical responsiveness. Due to the strong to excellent reliability, this version of the MQoL is useful in clinical as well as scientific setting.

## Introduction

The McGill-QoL Questionnaire (MQoL) is an example of a frequently used, generic questionnaire (Cohen et al., 1995; 1996; 1997; 2000; 2016). The questionnaire was originally developed for palliative patients; recently also used for chronic diseases. Breast cancer can be seen as a chronic condition due to the long-term medical follow- up (Ganz et al., 1993). The questionnaire counts 16+1 questions, which relate to the following domains: physical symptoms; physical wellbeing, psychological symptoms; existential wellbeing and support. A Likert scale with 11 possibilities (0-10) is used for the 16 questions and part D is an open question. The advantage of using a Likert scale is the fact that it is a multidimensional scale using different statements whereby respondents are asked to indicate which statement is most appropriate to their individual situation. The more statements are given, the more detailed the answer of the respondent can be given and the less ceiling- and floor effects can be obtained. The MQoL can be completed in ± 7 minutes. An important advantage of using of this questionnaire is the fact that it is an existential questionnaire. Hence, patients can indicate up to three physical complaints or problems, of which they experience the most burden and influence on personal wellbeing. This is an important advantage in breast cancer characterized by different post treatment morbidity. Since the development of the MQoL by Cohen et al. different clinical studies have demonstrated that the English version is valid, reliable and acceptable for the measurement of the QoL in patients with cancer and has good responsiveness as well (Cohen et al., 1995; 1996; 1997; 2000).

The MQoL assesses the quality of life in patients that is present at a given time and is already translated into several languages, including Dutch. Research into the measurement properties of the Dutch version is lacking. The purpose of this cross- sectional validation study is to assess the validity, reliability and responsiveness of the Dutch MQoL.

## Methods

### Patients

Based upon a sample size calculation with alpha = .05 / power = .80 / correlation >.5 and taking into account a drop-out rate of 10%, a sample size of 25 breast cancer patients needed to be recruited.

Patients from the Antwerp University Hospital;,Interdisciplinary Breast Clinic were recruited in March and April 2015 (n = 26). All subjects who met the following inclusion criteria were eligible: 1) patients with a history of breast cancer whom had finished their initial treatment (except: hormone or targeted therapy), 2) patients’ age was 18 or older, 3) patients had to be Dutch speaking. Patients who had extensive metastases or recurrence of (breast) cancer were excluded.

### Recruitment and data collection

Eligible patients were contacted by telephone to invite them for participation. After providing informed consent the patients filled out the questionnaire three times. First, a set of questionnaires needed to be filled out in the hospital. There, patients independently completed the EORTC QLQ-C30 (Aaronson et al., 1993; Sprangers et al., 1998; Fayers et al., 2002), Symptom Scales and MQoL-Dutch Questionnaire (www.kenniscentrapalliatievezorg.nl). Additionally the patients answered 3 questions concerning the MQoL: (1) Was each question understandable? (2) Was the scoring system clear? and (3) Were all questions related to your current health situation? If a patient answered “No”, a further explanation was asked. Second, patients received an envelope with a blank copy of MQoL questionnaire, which needed to filled out 24-48h after the first set of questionnaires. Patients were asked to send back the pre-stamped envelope within two weeks. Third, patients who completed all questionnaires were contacted again after an average time span of 7 months (n = 26). Aside the MQoL, patients also scored the Global Rating Scale of Perceived Effect (GRSPE) (Kamper, Maher et al., 2009).

### Data analysis

To perform statistical analyses, SPSS Statistics® version 22.0 (IBM, USA) for Macintosh/Windows was used. The socio-demographic data and scores were analysed descriptively. Normality distribution was assessed with One-Sample Kolmogorov- Smirnov test and Levene’s test. To evaluate validity (construct, content), convergence was tested between EORTC QLQ-C30 and MQoL. Pearson correlation coefficients for normal distributed variables and Spearman rank correlation coefficients for variables that were non-normally distributed were calculated. Internal consistency of the questionnaire was calculated using Cronbach's α. To evaluate the difference (p > .05) between the results of two MQoL scores, a paired sampled t-test was used. Test-retest reliability was analysed using Intraclass Correlation Coefficients (ICC(1,1)). Distribution- based measures which were used to determine important change, were the S.E.M. (standard error of measurements) and the MDC (minimal detectable change) for all subdomains of the MQoL, as well as for the total score, with following formula: S.E.M. = SD √(1-ICC). Calculation of the MDC is necessary to evaluate clinically important changes, using the formula: MDC = 1.96 x SEM x √2, with a conventional confidence level of 95% (Devoogdt et al., 2011). Comparisons were done between the MQoL administered in the hospital (MQoL1) and the first time at home (MQoL2), as well as between the second (MQoL3) and the first time at home.

### Ethical Approval

This study was approved by the Ethical Committee of the Antwerp University Hospital (UZA) and University of Antwerp, the Belgian registration number is B300201523253.

## Results

Thirty-five patients were contacted, of which 30 agreed to participate to the study. Table I summarizes the characteristics of all included patients. There were 2 dropouts between MQoL2 and MQoL3. (Table I)

**Table I:** — Characteristics of the 26 included patients

Parameter	Outcome
Age (years) (Mean ± SD)	63 ± 11
Weight (kg) (Mean ± SD)	73.7 ± 14.6
Height (m) (Mean ± SD)	1.64 ± 0.04
BMI (kg/m2) (Mean ± SD)	27.3 ± 5
Follow-up (months) (Med.; IQR)	39; 33
Marital status (n, %)	Single: 4 (15%) Cohabiting: 22 (85%)
Localization of tumour (n, %)	Right: 12 (46%) Left: 14 (54%)
Type of operation (n, %)	Tumourectomy: 14 (54%) Mastectomy: 12 (46%)
SLNB vs. ALND (n, %)	SLNB: 24 (92%) ALND: 2 (8%)
Radiotherapy (n, %)	Yes: 25 (96%) No: 1 (4%)
Chemotherapy (n, %)	None: 16 (61.5%) Neo-adjuvant: 3 (11.5%) Adjuvant: 7 (27%)
Target therapy (n, %)	Yes: 1 (4%) No: 25 (96%)
Hormonal therapy (n, %)	Yes: 20 (77%) No: 6 (23%)
Mean values total score MQoL: MQoL1 (Mean ± SD) MQoL2 (Mean ± SD) MQoL3 (Mean ± SD)	6.87 ±1.77 6.9 ± 1.56 7.5 ±1.56

(n = number, SD = standard deviation, kg = kilogram, m = meter, m2 = square meter, BMI = body mass index, Med = median, IQR = inter quartile range, SLNB = sentinel lymph node biopsy, ALND = axillary lymph node dissection, MQoL = McGill quality of life questionnaire)

### Validity

All patients completed the questionnaire about the content validity of the MQoL-Dutch version. All patients mentioned that (1) the questions, as well as (2) the scoring system was clear. 100% answered “Yes” on both questions. Only 2 patients (7,7%) answered “No” to the third question (relevance of the questionnaire to the current health situation), merely due to the time aspect between treatment and moment of the interview.

Table II presents the correlations between the MQoL domains and the different subscales of the EORTC QLQ-C30.

### 

**Table II:** — Clinimetric properties of the MQoL-Dutch version

**CLINIMETRICS MQOL-DUTCH version**				
Validity* (p < 0.05)	Convergent (EORTC- QLQ-C30)	Physical symptoms: 0.76^a^
		Physical well-being: 0.75^b^
		Psychological symptoms: 0.74^c^
		Existential well-being: 0.71^d^
		Total score: 0.66^e^
**Reliability (p < 0.05)**	Test-retest reliability (ICC _(1,1)_, 95%CI)		**MQoL 1-2**	
		Physical symptoms	0.91 (0.75-0.96)	
		Physical well-being	0.68 (0.40-0.85)	
		Psychological symptoms	0.82 (0.65-0.92)	
		Existential well-being	0.86 (0.72-0.94)	
		Support	0.92 (0.84-0.96)	
		Total score	0.93 (0.85-0.97)	
	Internal Consistency (Cronbach’s α)		**MQoL 1-2**	
		Physical symptoms	0.95	
		Physical well-being	0.82	
		Psychological symptoms	0.91	
		Existential well-being	0.93	
		Support	0.96	
		Total score	0.96	
**Responsiveness**	SEM		**MQoL 1-2**	**MQoL 2-3**
		Physical symptoms	0.96	1.59
		Physical well-being	1.15	1.04
		Psychological symptoms	0.93	1.82
		Existential well-being	0.68	1.19
		Support	0.53	1.11
		Total score	0.44	0.83
	MDC		**MQoL 1-2**	MQoL 2-3
		Physical symptoms	2.66	4.41
		Physical well-being	3.19	2.88
		Psychological symptoms	2.58	5.05
		Existential well-being	1.89	3.30
		Support	1.47	3.08
		Total score	1.22	2.30

* = Pearson correlations for normally distributed variables Note^1^: Correlations between EORTC QLQ-C30 and MQoL, where: ^a^ is correlated with PFS (physical functioning score); ^b^ is correlated with RFS (role functioning score); ^c^ is correlated with EFS (emotional functioning score); ^d^ is correlated with CFS (cognitive functioning score); and ^e^ is correlated with QoLS (Quality of Life score) Note^2^: Cut off values used for correlation r: 0-0.2 = no correlation, 0.2-0.4 = weak, 0.4-0.6 = moderate, 0.6-0.8 = strong, 0.8-1 = very strong (McDowell, 2006; Taylor, 1990) Note^3^: Cut off values used for ICC: < 0.4 = weak; 0.4-0.75 = moderate; 0.75-0.9 = strong; > 0.9 = very strong (Fleiss, 2011; McDowell, 2006) Note^4^: Cut off values used for Cronbach's α : α < 0.5 = unacceptable, 0.5 ≤ α > 0.6 = weak, 0.6 ≤ α < 0.7 = acceptable, 0.7 ≤ α < 0.9 = good, α ≥ 0.9 = excellent (Bland & Altman, 1997; McDowell, 2006)

### Reliability

The paired sampled t-test showed no statistical significant difference between the two Dutch MQoL-total scores, as well as between the scores of several domains on two different time points. For this reason it can be assumed there was no clinical change for the patients. ICC's, 95% Confidence Interval with level of significance and internal consistency using Cronbach's α are shown in Table II. Previous studies have shown good clinimetrical properties (i.e. internal consistency, test-retest reliability, concurrent and construct validity) for the English version of the questionnaire. These properties appear to correlate well with clinimetrical characteristics shown in the MQoL-Dutch version (Cohen et al., 1995; Cohen and Mount, 2000).

### Responsiveness

A very strong correlation of 0.93 between MQoL1 and MQoL2 was measured. A strong correlation was seen between MQoL2 and MQoL3 (0.77) of which we can conclude our population remained relatively stable within a period of seven months.

Mean total score for MQoL3 is 7.5 and for MQoL2 6.9. Standard deviations (SD’s) for both moments in time are 1.56.

In Table II variability between MDC’s can be noticed amongst different subdomains of the MQoL. Despite that, a MDC in total score of only 1.22 (12%) can be seen, what is needed to detect a factual change within a patients’ QoL. The MDC value after the third conduction of the MQoL was slightly higher than after the second.

## Discussion

From the obtained results, we can conclude that the Dutch version of the MQoL is valid and reliable. Also, a statistical responsiveness was determined. The results of the current study compare well with findings of previous studies concerning the clinimetric data of the original version of the MQoL (Cohen et al., 1995, Cohen and Mount, 2000, Faria and Eluf-Neto, 2014).

Focussing on the convergent validity within this study, it is clear that the subdomain ‘existential wellbeing’ has the strongest correlation with the subdomain ‘general QoL’ of the EORTC QLQ-C30 (r = 0.71). Previously Cohen et al. concluded that questions based on existential wellbeing are an important determinant for the overall QoL, in which they can reflect the subjective wellbeing of an individual (Cohen et al., 1995; 1996; 1997).

In comparison with the results of Faria and Eluf-Neto (2014) about the test-retest reliability, all ICC- values are systematically higher in our study. In parallel with the study of Cohen and Mount (2000) we can see that our values are quite similar (ICC total score MQoL = 0.93 vs. 0.75 with a range for the subdomains going from 0.68 to 0.92 vs. 0.62 to 0.85) When analysing the ICC-values within our study, we note that the subdomain ‘physical well- being’ has a moderate score relative to the strong to excellent scores of the other four subdomains of the MQoL. A possible explanation is the mental state of the patient, which varies daily and has an enormous influence on the subjective sensation of physical wellbeing (Cohen and Mount 2000). The same occurs in the study of Faria and Eluf-Neto (2014) whereby the ICC-value for this subdomain is lower in comparison with the other subdomains. The former study also reported a good internal consistency in the several subdomains (α = 0.75 tot 0.84). When the comparison is made with our study, the values vary from poor ('support' α = 0.60) to excellent ('physical symptoms' α = 0.97).

From the obtained results of this study, we can only make a statement regarding the statistical responsiveness of the MQoL in a Dutch-speaking breast cancer population. With a MDC of 1.22 points in total score; a real change in a patients’ QoL can be assumed. We were unable to compare our results with other studies regarding MDC.

In our study, we cannot report about clinical responsiveness, because none of the patients received an intervention and our group remained too stable. To examine this part of responsiveness a prospective study is needed, where patients are followed during the entire period of treatment.

## Conclusion

The Dutch version of the MQoL is a valid and reliable questionnaire, with an important convergent validity for parallel subdomains. Likewise, the MQoL generated reliable results for breast cancer patients and shows statistical responsiveness. With a MDC of 1.22 points in total score; a factual change is demonstrated. Due to these excellent psychometric properties, the Dutch version of the MQoL is useful assessing breast cancer patients’ QOL.
